# Altered amyloid-β structure markedly reduces gliosis in the brain of mice harboring the *Uppsala APP* deletion

**DOI:** 10.1186/s40478-024-01734-x

**Published:** 2024-02-05

**Authors:** María Pagnon de la Vega, Stina Syvänen, Vilmantas Giedraitis, Monique Hooley, Evangelos Konstantinidis, Silvio R. Meier, Johanna Rokka, Jonas Eriksson, Ximena Aguilar, Tara L. Spires-Jones, Lars Lannfelt, Lars N. G. Nilsson, Anna Erlandsson, Greta Hultqvist, Martin Ingelsson, Dag Sehlin

**Affiliations:** 1 Department of Public Health and Caring Sciences, Geriatrics, Uppsala University, Uppsala, Sweden; 2 UK Dementia Research Institute, Edinburgh Medical School, University of Edinburgh, Edinburgh, UK; 3 Centre for Discovery Brain Sciences, University of Edinburgh, Edinburgh, UK; 4 Department of Medicinal Chemistry, Division of Organic Pharmaceutical Chemistry, Uppsala University, Uppsala, Sweden; 5 PET Centre, Uppsala University Hospital, Uppsala, Sweden; 6 BioArctic AB, Stockholm, Sweden; 7 Department of Pharmacology, University of Oslo and Oslo University Hospital, Oslo, Norway; 8 Department of Pharmacy, Uppsala University, Uppsala, Sweden; 9 Krembil Brain Institute, University Health Network, Toronto, ON Canada; 10 Tanz Centre for Research in Neurodegenerative Diseases, Departments of Medicine and Laboratory Medicine and Pathobiology, University of Toronto, Toronto, ON Canada

**Keywords:** Alzheimer’s disease (AD), Amyloid precursor protein (APP), Amyloid-beta (Aβ), PET imaging, Microglia, Astrocytes, Immunotherapy

## Abstract

Deposition of amyloid beta (Aβ) into plaques is a major hallmark of Alzheimer’s disease (AD). Different amyloid precursor protein (*APP*) mutations cause early-onset AD by altering the production or aggregation properties of Aβ. We recently identified the *Uppsala APP* mutation (*APPUpp*), which causes Aβ pathology by a triple mechanism: increased β-secretase and altered α-secretase APP cleavage, leading to increased formation of a unique Aβ conformer that rapidly aggregates and deposits in the brain. The aim of this study was to further explore the effects of *APPUpp* in a transgenic mouse model (tg-UppSwe), expressing human *APP* with the *APPUpp* mutation together with the *APPSwe* mutation. Aβ pathology was studied in tg-UppSwe brains at different ages, using ELISA and immunohistochemistry. In vivo PET imaging with three different PET radioligands was conducted in aged tg-UppSwe mice and two other mouse models; tg-ArcSwe and tg-Swe. Finally, glial responses to Aβ pathology were studied in cell culture models and mouse brain tissue, using ELISA and immunohistochemistry. Tg-UppSwe mice displayed increased β-secretase cleavage and suppressed α-secretase cleavage, resulting in AβUpp42 dominated diffuse plaque pathology appearing from the age of 5–6 months. The γ-secretase cleavage was not affected. Contrary to tg-ArcSwe and tg-Swe mice, tg-UppSwe mice were [^11^C]PiB-PET negative. Antibody-based PET with the 3D6 ligand visualized Aβ pathology in all models, whereas the Aβ protofibril selective mAb158 ligand did not give any signals in tg-UppSwe mice. Moreover, unlike the other two models, tg-UppSwe mice displayed a very faint glial response to the Aβ pathology. The tg-UppSwe mouse model thus recapitulates several pathological features of the *Uppsala APP* mutation carriers. The presumed unique structural features of AβUpp42 aggregates were found to affect their interaction with anti-Aβ antibodies and profoundly modify the Aβ-mediated glial response, which may be important aspects to consider for further development of AD therapies.

**Supplementary Information:**

The online version contains supplementary material available at 10.1186/s40478-024-01734-x.

## Introduction

Accumulation of amyloid beta (Aβ) as plaques and tau as tangles, accompanied by various degrees of astrocytosis and microgliosis, are the main pathological hallmarks in the Alzheimer’s disease (AD) brain. The Aβ peptide spontaneously aggregates into different structures that differ not only in their aggregation states, but also in their impact on the surrounding brain milieu. Among such Aβ species, oligomers and protofibrils are considered to be the most toxic forms that drive neurodegeneration in AD (reviewed in [17]). Studies on familial AD (FAD) mutations have provided a mechanistic understanding of Aβ generation and deposition. For example, the *APPSwe* mutation promotes β-secretase cleavage and thereby increased formation of Aβ [25], while the *APPArc* mutation leads to a conformational change of the Aβ peptide (AβArc) that facilitates the formation of Aβ oligomers and protofibrils. Further, *APPLon* and other mutations located close to the C terminal end of the Aβ-domain in APP favor generation of longer and more aggregation prone Aβ variants. Apart from affecting Aβ production, FAD mutations can also influence the fibrillar structure of Aβ deposits [41].

We recently discovered a new *APP* mutation, *APPUpp*, a six amino acid (aa) deletion within the Aβ peptide which leads to higher levels of Aβ by an increased β-secretase cleavage and a shifted location of α-secretase cleavage. In addition, this deletion promotes rapid fibrillization of mutated Aβ42 (AβUpp1-42_Δ19–24_) that deposits as unique polymorphs in the affected brain [27]. Thus, this is the first example of an *APP* mutation that causes AD via several pathogenetic mechanisms.

Further insights into the pathophysiological mechanisms of Aβ aggregation and deposition can be gained by in vivo investigations of animal models expressing human *APP* with FAD mutations [34]. We have previously generated the tg-ArcSwe and tg-Swe lines, both under the Thy-1 promoter [20], that display various features of Aβ brain pathology. Tg-ArcSwe mice, with the *APPSwe* mutation for increased Aβ production and the *APPArc* for increased Aβ aggregation, display early deposition of Aβ plaques composed of fibrils with a structure similar to that found in the brains of sporadic AD patients [20, 29, 42]. On the other hand, tg-Swe mice that express only wild-type Aβ (Aβwt) have a later plaque deposition, starting at the age of 10–12 months, with somewhat more loosely structured plaques composed of fibrils of the same structure as in some forms of FAD-related Aβ brain deposits [20, 29, 42]. Moreover, both these and other FAD models feature activation of glial cells as a response to the Aβ pathology. Both astrocytes and microglia are closely associated with Aβ plaques in the models and are presumably involved both in Aβ elimination and in formation of the dense core of amyloid plaques. The impact of glial cells in AD pathology has therefore recently received much attention.

Apart from explaining pathological mechanisms, *APP* transgenic mice have proven useful for the development of novel therapeutic and diagnostic approaches. As an example, the tg-ArcSwe model was used for the preclinical efficacy studies of mAb158, the murine parent of the anti-Aβ monoclonal antibody lecanemab, which selectively recognizes soluble Aβ protofibrils [2]. Lecanemab was recently approved by the US Food and Drug Administration (FDA) for treatment of AD after a successful phase III clinical trial [40].

In the present study, we developed a new mouse model (tg-UppSwe), with transgenic expression of *APPUpp,* combined with *APPSwe*, to further explore the pathogenetic effects of the *APPUpp* mutation, both in vivo and ex vivo. We hypothesized that the altered APPUpp processing and rapid aggregation of AβUpp would resemble that of human *APPUpp* mutation carriers [27], leading to specific pathological features of this mouse model. Mainly, we found that tg-UppSwe mice display aggressive Aβ aggregation, leading to widespread and abundant diffuse plaque pathology with a distinct structure that is differentially detected by antibodies in vivo and does not activate glial cells. These findings provide important insights into the mechanisms of Aβ related pathology and may guide future development of therapies against AD.

## Methods

### Animals

To generate the tg-UppSwe mouse line, cDNA of human *APP* (*hAPP*) with the *APPSwe* (*KM670/671NL*) [25] and the *APPUpp* (*690-695Δ*) [27] mutations was inserted into the murine Thy-1323-cassette, as previously described for the generation of the tg-Swe and tg-ArcSwe mouse lines [20]. Tg-UppSwe mice will therefore be fully comparable to tg-ArcSwe and tg-Swe in terms of any potential promoter induced effects. The linearized DNA was injected into oocytes at the Karolinska Center for Transgene Technologies (KCTT, Karolinska Institute, Stockholm, Sweden), resulting in nine founders of both sexes. The tg-UppSwe mouse line was established from one male founder by heterozygous breeding on a C57/BL6J-BomTac background. Upon further breeding, mice of both sexes from generation 6–8 were used in the study. For comparison, sex- and age-matched wildtype (wt) littermates were included. Presence of the human transgene was confirmed by PCR, using two sets of primer pairs that framed the Thy-1 basal promoter region and the *APP* coding region. Copy numbers of the human *APP* gene inserted in the mouse genome were assessed using TaqMan real-time PCR. In short, DNA was extracted from mouse brain tissue using DNeasy Blood & Tissue Kit (Qiagen, Germany). Taqman human APP assay Hs01255859_cn, together with an internal copy number reference (mouse transferrin receptor gene, *Tfrc*), was used according to the manufacturer’s instructions (Thermo Fisher Scientific, USA). Quantitative PCR (qPCR) was performed on the StepOnePlus™ Real-Time PCR System and results were analyzed by the CopyCaller v2.1 software (Thermo Fisher Scientific, USA). Tg-UppSwe mice were compared with tg-ArcSwe mice, expressing *hAPP* with the *APPArc* (E693G) and *APPSwe* mutations, and with tg-Swe mice, harboring only the *APPSwe* mutation [20]. In addition, wt mice were used for generation of primary cell cultures. All mice were bred on a C57/BL6J-BomTac background. The total number of animals included in the study is given in Table 1. All procedures were approved by the Stockholm North or Uppsala County Animal Ethics boards (N174-15, C85-16, 5.8.18-13350/17, 5.8.18-20401/20), following the rules and regulations of the Swedish Animal Welfare Agency, and were in compliance with the European Communities Council Directive of 22 September 2010 (2010/63/EU).

**Table 1:** Number of animals used in the study

Age (months)	tg-UppSwe	tg-ArcSwe	tg-Swe	wt	total
4–6	10	–	–	3	13
8–10	10	–	–	–	10
13–14	10	–	–	–	10
17–19	35	9	9	9	62
Total	65	9	9	12	95

### Brain tissue homogenization and Aβ ELISAs

Fresh frozen brain tissue (cerebrum) was homogenized at a 1:5 tissue:buffer ratio, using a Precellys Evolution homogenizer (Bertin Technologies**,** Montigny-Le-Bretonneux, France) to sequentially extract TBS, TBS with 0.5% Triton X-100 (TBS-T) and formic acid (FA) soluble fractions of Aβ. After homogenization in TBS, samples were centrifuged at 16,000× *g* for 1 h at 4 °C (TBS_16K_). For a subset of experiments, a fraction of this supernatant was further centrifuged at 100,000× *g* followed by collection of the supernatant (TBS_100K_). Homogenization of the remaining tissue pellet was repeated according to the same procedure with TBS-T, then followed by FA (Additional file 1: Fig. S1).

Brain extracts were analyzed with Aβ1-40 and Aβ1-42 ELISAs, as previously described [27]. In brief, 96-well half-area plates were coated over night with 50 ng/well of anti-Aβ40 (custom production, Agrisera) or anti-Aβ42 (700254, Thermo Fisher Scientific, USA), then blocked with 1% BSA in PBS for 3 h at RT. TBS_16K_, TBS-T and FA brain extracts were diluted in ELISA incubation buffer (PBS, 0.1% BSA, 0.05% Tween) and incubated over night at 4 °C, followed by detection with 0.5 µg/ml biotinylated 3D6 and HRP-conjugated streptavidin (Mabtech AB, Nacka, Sweden). Signals were developed with K Blue Aqueous TMB substrate (Neogen Corp., Lexington, KA, US). Plates were developed and read with a spectrophotometer at 450 nm.

The TBS and TBS-T extracts were also analyzed for soluble Aβ aggregates using two different sandwich ELISAs. The first, based on 3D6 as both capture and detection antibody, allows for detection of Aβ aggregates from the size of a dimer, but not for monomeric Aβ. The second preferentially detects larger soluble Aβ aggregates as it utilizes the Aβ protofibril selective antibody mAb158 for capture and 3D6 for detection [23]. Ninety-six-well half-area plates were coated over night with 3D6 (50 ng/well) and blocked with 1% BSA in PBS for 3 h at RT. Brain extracts were diluted in ELISA incubation buffer (PBS, 0.1% BSA, 0.05% Tween) and incubated over night at 4 °C, followed by 3D6-biotin detection and development as above.

### Immunohistochemistry and thioflavin S staining

Right hemispheres from fresh frozen mouse brains were sectioned at 20 µm. Next, the sections were fixed with 4% paraformaldehyde and treated with pre-heated citrate buffer, pH 6.3, for 30 min followed by 70% formic acid for 5 min. Aβ was visualized with anti-Aβ40 (Agrisera, Umeå, Sweden), anti-Aβ42 1:1000 (700254, Thermo Fisher Scientific, USA), mAb158 or 3D6 1:1000 (in-house expression); activated astrocytes with anti-GFAP 1:200 (Abcam, Cambridge, UK); and microglia with antibodies against Iba1 (1:200) (Wako chemicals, Richmond, VA) and TREM2 1:200 (AF1729; R&D, Abingdon, UK). For colorimetric staining the Vector NovaRED™ horse radish peroxidase (HRP) substrate kit (Vector Laboratories, Burlingame, CA) was used for detection while for fluorescent staining Alexa secondary antibodies were used (Thermo Fisher Scientific, USA). For thioflavin S (ThS) staining, sections were pretreated in 95% and 70% ethanol (3 min in each), and quickly rinsed in water before they were incubated in 0.1% ThS for 10 min. Finally, the sections were briefly washed in 80% ethanol and water, dehydrated in ethanol, cleared in xylene and mounted with DPX.

### Array tomography

Fresh brain tissue was collected from an 18-month-old tg-UppSwe mouse [11, 14]. Small tissue blocks containing cortex were fixed in 4% paraformaldehyde and 2.5% sucrose in 20 mM phosphate buffered saline pH 7.4 (PBS) for 3 h. Samples were dehydrated through cold graded ethanol of ascending strengths and embedded into LR White Resin (Electron Microscopy Sciences, Hatfield, PA, USA), which was allowed to polymerize overnight at 53 °C. Resin embedded tissue blocks were cut into array ribbons of 70 nm thick sections using an ultracut microtome (Leica, Wetzlar, Germany) equipped with a Jumbo Histo Diamond Knife (Diatome, Hatfield, PA) and collected onto gelatin coated coverslips. For detection of colocalization between pathological proteins and synapses, array ribbons were immunostained with a primary antibody against post-synapses (PSD95), pre-synapses (synaptophysin) and with the OC polyclonal antibody against fibrillar Aβ [12]. Sections were counterstained with 0.01 mg/mL 4′-6-diamidino-2-phenylindole (DAPI). For each experiment, a short extra ribbon was used as a negative control. Images were obtained on serial sections using an AxioImager Z2 epifluorescent microscope (Carl Zeiss, Oberkochen, Germany) with a 10× objective for tile scans and 63× 1.4 NA Plan-Apochromat objective for high resolution images. Images were acquired with a CoolSnap digital camera and AxioImager software with array tomography macros (Carl Zeiss). Images from each set of serial sections were converted into image stacks and aligned using the ImageJ plug-in, MultiStackReg (courtesy of Brad Busse and P. Thevenaz, Stanford University) [39]. Regions of interest within the cortical neuropil were chosen (10 μm^2^) and their proximity to plaque edges recorded (< 20 μm from a plaque edge considered “near” plaques and > 20 μm from a plaque edge considered “far” from plaques). Image stacks were then binarised using thresholding algorithms in ImageJ. For synaptic staining, image stacks were binarised using an ImageJ script that combines different thresholding algorithms in order to select both high and low intensity synapses in an automated and unbiased manner. To examine pathological protein presence at the synapse, thresholded images were processed and analyzed in MATLAB to remove background noise.

### Antibody production and radiochemistry

For PET imaging with antibody-based ligands (immunoPET), the bispecific brain penetrating Aβ antibodies RmAb3D6-scFv8D3 [5] and RmAb158-scFv8D3 [9] were used. While 3D6 binds to the N-terminus of Aβ [1], mAb158 preferentially binds to soluble Aβ protofibrils and to some extent also to Aβ fibrils [2]. Both bispecific antibodies actively enter the brain via receptor mediated transcytosis, using the TfR binding domain scFv8D3. The antibodies were produced recombinantly in Expi293 cells, according to previously described procedures [4] and radiolabeled with iodine-124 (^124^I) for PET imaging or with iodine-125 (^125^I) for ex vivo studies [36]. In brief, for ^124^I-labeling, a [^124^I]iodide stock solution (Advanced Center Oncology Macerata, Montecosaro, Italy) was pre-incubated for 15 min with half a volume of 50 µM NaI and then neutralized with 0.5% HAc and PBS. After adding 90 µg antibody, the reaction was initiated by the addition of 40 µg Chloramine-T (Sigma Aldrich, Stockholm, Sweden) and, after 120 s, quenched with 80 µg sodium metabisulfite (Sigma Aldrich). For ^125^I labeling, a [^125^I]iodide stock solution (PerkinElmer Inc., Waltham, MA, USA) was mixed with 40 µg of antibody in PBS and the reaction was initiated by the addition of 5 µg Chloramine-T (Sigma Aldrich) and, after 90 s, quenched with 10 µg sodium metabisulfite (Sigma Aldrich). The radiolabeled antibody was purified from non-reacted [^125^I]iodide with Zeba spin desalting columns (7K MWCO, 0.5 mL, ThermoFisher, Uppsala, Sweden). The molar activity of [^124^I]RmAb3D6-scFv8D3 and [^124^I]RmAb158-scFv8D3 was 135 MBq/nmol and 133 MBq/nmol, respectively.

The amyloid PET radioligand [^11^C]PiB, formulated in 10% ethanol in PBS, was synthesized as previously described with minor modifications to adapt the procedure to our in-house built synthesis device (TPS) [13].

### PET imaging

Eighteen months old tg-UppSwe, tg-ArcSwe, tg-Swe and wt mice underwent PET imaging with [^11^C]PiB or with either of the two antibody radioligands, [^124^I]RmAb3D6-scFv8D3 or [^124^I]RmAb158-scFv8D3 (n = 3 per ligand and genotype). For [^11^C]PiB-PET, mice were injected with 13.2 ± 2.9 MBq radioligand and PET data acquired between 40–60 min after injection was used for all subsequent analyses. For immunoPET, mice were given 0.2% NaI in the drinking water to reduce thyroidal uptake of ^124^I. The following day, the mice were injected with 8.7 ± 1.6 MBq of [^124^I]RmAb3D6-scFv8D3 or [^124^I]RmAb158-scFv8D3, corresponding to an antibody dose of 2.3 nmol/kg body weight. Four days after antibody injection, mice were PET scanned for 60 min with either a Triumph Trimodality System (TriFoil Imaging, Chatsworth, CA) or a nanoScan system PET/MRI (Mediso, Budapest, Hungary). PET scans performed with the Triumph system were reconstructed with a 3-dimensional ordered-subsets expectations maximization, with 20 iterations. The PET data acquired with the Mediso system were reconstructed using a Tera-TomoTM 3D algorithm (Mediso) with four iterations and six subsets. Each mouse underwent a CT scan following PET. All subsequent image processing was performed with Amide version 1.0.4. The CT and PET data were manually aligned with a T2-weighted mouse brain atlas [22] for quantification of activity in the cerebrum.

### Single injection immunotherapy and brain distribution

To investigate potential acute treatment effects and to further assess brain distribution of the two antibodies used for immunoPET, 18-months-old tg-UppSwe mice were injected with PBS (n = 4), or with a therapeutic dose (32 nmol/kg) of RmAb3D6-scFv8D3 (n = 5) or RmAb158-scFv8D3 (n = 5). The bispecific antibody preparations were supplemented with trace amounts (1.2 nmol/kg; 18 ± 1.6 MBq/kg) of [^125^I]RmAb3D6-scFv8D3 or [^125^I]RmAb158-scFv8D3 for quantification. After three days, the mice were euthanized by intracardiac perfusion. Radioactivity was quantified in brain and blood as well as in TBS_16K_, TBS-T and FA extracts of homogenized brain.

### Ex vivo antibody brain distribution

After immunoPET imaging or administration of antibodies at a therapeutic dose, the mice underwent intracardiac perfusion with 20 ml 0.9% NaCl during 2.5 min. The brains were then isolated and separated into right and left hemispheres, followed by a further division of the left hemisphere into cerebrum and cerebellum. To assess the concentration of antibody in the brain tissue, radioactivity was quantified in the isolated brain regions using a gamma counter (Wizard 1480 Wizard™, Wallac Oy, Turku, Finland) and expressed as % of injected dose per gram brain tissue (%ID/g brain). To visualize brain distribution of radiolabeled antibody, 20 µm cryosections from the right hemisphere were exposed to phosphor imaging plates (MS, MultiSensitive, PerkinElmer, Downers Grove, IL) for 7 days. Plates were scanned with a Cyclone Plus phosphor imager (PerkinElmer, Waltham, MA) at 600 dpi resolution. Radioactivity distribution was visualized with ImageJ using a royal lookup table and combined with Aβ42 immunostaining of an adjacent brain section.

### Astrocyte cultures and Aβ uptake studies

To study interactions between Aβ and primary mouse astrocyte cultures, cerebral cortices of wt mice (n = 3) were dissected from embryonal day (E14) mice in Hank’s buffered salt solution (HBSS) supplemented with 50 U/ml Penicillin, 50 mg/ml Streptomycin, and 8 mM Hepes buffer (ThermoFisher Scientific). The cortices were centrifuged in fresh HBSS for 3 min at 150× *g* and then resuspended and dissociated into a homogenous solution. Any remaining blood vessels were allowed to sediment for 10 min. The supernatant was transferred to a new tube and centrifuged for 5 min at 150× *g*. The cell pellet was carefully resuspended in DMEM/F12 GlutaMax cell culture medium. The embryonic cortical stem cells were allowed to expand as neurospheres in DMEM/F12 GlutaMax medium (Invitrogen) supplemented with B27 (Invitrogen), 100 U/ml penicillin, 100 μg/ml streptomycin, 8 mM HEPES buffer, 10 ng/ml bFGF (Invitrogen, diluted in 10 mM Tris–HCl (pH 7.6) + 0.1% BSA and PBS) and 20 ng/ml EGF (BD biosciences), dissolved in MQ water). The cells were then dissociated and plated as a monolayer at a density of 3 × 10^4^ cells/cm^2^ on cover glasses coated with poly-L-ornithine and laminin. The following day, the growth factors were removed to start differentiation, resulting in co-cultures containing ~ 75% astrocytes after 7 days. Differentiated cell cultures were exposed to 0.1 μM Cy3-labeled sonicated fibrils of Aβ1-42_Upp_, Aβ1-42_Arc_ or Aβ1-42_wt_. Control cultures received culture medium without Aβ. After 24 h, the cells were washed three times in cell culture medium and the cover slips were transferred to new culture dishes.

### Preparation and Cy3 labeling of Aβ fibrils

To induce fibrillization, synthetic Aβ (200 µM in NaOH) was diluted in 2× PBS to a concentration of 100 µM and incubated on a shaker at 1500 rpm and 37 °C for 24 h. Tween-20 was then added to a final concentration of 0.01%.

For the labeling process, a Cy3^AM^ antibody labeling kit (GE Healthcare, PA33000) was used. The fibrils were gently mixed in the coupling buffer by vortexing and then supplemented with Cy3. The mixture was incubated for 1 h at RT in the dark, then purified from unreacted Cy3 with dialysis in PBS with 0.01% Tween-20 for 2 h. The resulting Cy3-Αβ fibrils were diluted in sterile PBS to a final concentration of 0.5 mg/ml and sonicated at 20% amplitude, 1 s on/off pulses for 1 min (#VCX130, Vibra Cell sonicator, Sonics, CT, USA).

### Immunocytochemistry

The cells were fixed for 15 min at RT with 4% paraformaldehyde, washed twice with PBS and permeabilized and blocked with 0.1% Triton X-100 (both from Sigma-Aldrich) and 5% normal goat serum (NGS, BioNordika) in PBS for 30 min at RT. Primary antibodies, diluted in 0.1% Triton X-100 with 0.5% NGS, were added and left to incubate for 1–4 h at RT or overnight at 4 °C. The cells were then washed 3 × 10 min with PBS before incubation with secondary antibodies (diluted in 0.1% Triton X-100 and 0.5% NGS) for 45 min at 37 °C or 1 h at RT. Cover slips were mounted onto microscope glass slides using VECTASHIELD hard set mounting medium with DAPI (DAKO). Imaging was performed using a Zeiss Observer Z1 Microscope, and the images were visualized with the Zen 2012 software and representative 40× images were captured. The primary antibodies used were chicken anti-Glial Fibrillary Acidic Protein (GFAP, 1:200, Abcam) and rabbit anti-lysosome-associated membrane protein-1 (LAMP-1, 1:200, Abcam). The secondary antibodies applied were AlexaFluor 488 (rabbit, 1:200, Thermofisher), and AlexaFluor 647 (chicken, 1:200, Thermofisher).

### GFAP ELISA

Quantification of GFAP in tg-UppSwe brain extracts was performed with a sandwich ELISA, as previously described [26]. Ninety-six-well half-area plates were coated over night with 25 ng/well of anti-GFAP antibody GA5 (Sigma Aldrich), then blocked with 1% BSA in PBS for 2 h at RT. The TBS_16K_ and TBS-T brain extracts were diluted in ELISA incubation buffer (PBS, 0.1% BSA, 0.05% Tween) and incubated over night at 4 °C, followed by 1 h incubation with 0.5 µg/ml polyclonal anti-GFAP (Dako, Z0334). Signals were detected with 0.5 µg/ml biotinylated goat anti-rabbit antibody in combination with HRP-conjugated streptavidin and K Blue Aqueous TMB substrate and read at 450 nm as above. A standard curve of recombinant GFAP (in-house produced [24]) was used for quantification.

### TREM2 ELISA

Soluble TREM2 (sTREM2) was detected with a sandwich ELISA, performed in a similar manner as for the assays described above. Ninety-six-well half-area plates were coated over night with 25 ng/well of anti-TREM2 antibody AF1729 (R&D, Abingdon, UK), then blocked with 1% BSA in PBS for 2 h at RT. The TBS_16K_ and TBS-T brain extracts were diluted in ELISA incubation buffer (PBS, 0.1% BSA, 0.05% Tween) and incubated over night at 4 °C, followed by detection with 0.25 µg/ml biotinylated anti-TREM2 BAF1729 (R&D), HRP-conjugated streptavidin and K Blue Aqueous TMB substrate and read at 450 nm with a spectrophotometer. A standard curve of recombinant TREM2 was used for quantification.

### Statistics

Data was analyzed using GraphPad Prism (version 6 and 7, San Diego, CA). Comparisons of three or more groups were analyzed by one-way ANOVA for single datasets and by two-way ANOVA for multiple datasets, followed by Tukey’s post hoc test. A *p* value threshold of 0.05 was used for assessment of the statistical significance. Values are shown as means ± SD.

## Results

### Aβ production and deposition

**Fig. 1:**
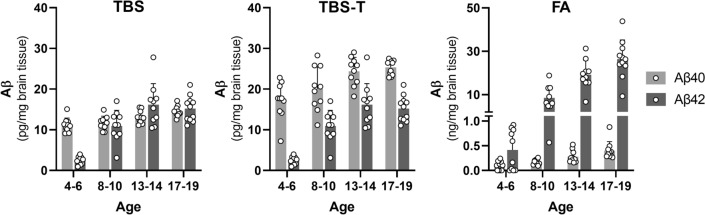
Brain Aβ concentration in tg-UppSwe mice. Aβ1-40 and Aβ1-42 concentrations in tg-UppSwe mice of different ages (n = 10 per group, equal sex distribution), measured in brain homogenates sequentially extracted with TBS, TBS-triton (TBS-T) and formic acid (FA). NB the different units on the y-axes

Transgenic mice, tg-UppSwe, were designed to express human *APP* (*hAPP*) with the *APPUpp* (*690-695Δ*) mutation [27] in combination with the *APPSwe* mutation (*KM670/671NL*), to increase the overall Aβ production [20, 25]. Quantitative PCR analysis showed that a single copy of *hAPP* had been inserted in the tg-UppSwe genome (analysis range 0.87–2.05), which is low compared to other APP-transgenic models. For example, tg-Swe and tg-ArcSwe mice that have a similar genetic design were found to harbor six and two *hAPP* copies, respectively (Additional file 1: Fig. S2A), which is consistent with their previously reported APP protein expression [21]. To investigate if the tg-UppSwe mice showed alterations in APP processing as previously found in the human *APPUpp* mutation carriers [27], α- and β-secretase cleavage of APP were investigated in brain tissue extracts. Consistent with analyses of human *APPUpp* carriers, tg-UppSwe mice displayed a relative reduction in the soluble APPα fragment (sAPPα), which is the fragment resulting from alpha-secretase cleavage of APP, as well as an increase in the soluble APPβ fragment (sAPPβ), the fragment resulting from beta-secretase cleavage of APP (Additional file 1: Fig. S2B–D). Tg-UppSwe mice showed no difference in survival and displayed similar body weights as wt mice (Additional file 1: Fig. S3). To study production and deposition of Aβ in tg-UppSwe mice, sequentially extracted brain tissue samples were analyzed with ELISA. In 4–6 months-old mice, the TBS fraction, which reflects soluble Aβ, contained five- to ten-fold more Aβ1-40 compared to Aβ1-42. The levels of Aβ1-40 increased slightly with age, while Aβ1-42 levels showed a more pronounced increase (Fig. 1). The TBS-T fraction, reflecting membrane bound Aβ, displayed a pattern similar to TBS, but with slightly higher levels of both Aβ1-40 and Aβ1-42. In the FA fraction, representing insoluble brain Aβ (including fibrils in plaques) tg-UppSwe brain displayed low concentrations of Aβ1-40, with only a small increase with age, suggesting a very low degree of Aβ1-40 deposition. In contrast, Aβ1-42 increased dramatically from the youngest age group (4–6 months) to the next age-group and then further as the mice aged, finally displaying an almost 100-fold higher concentration compared to Aβ1-40 (Fig. 1).

**Fig. 2:**
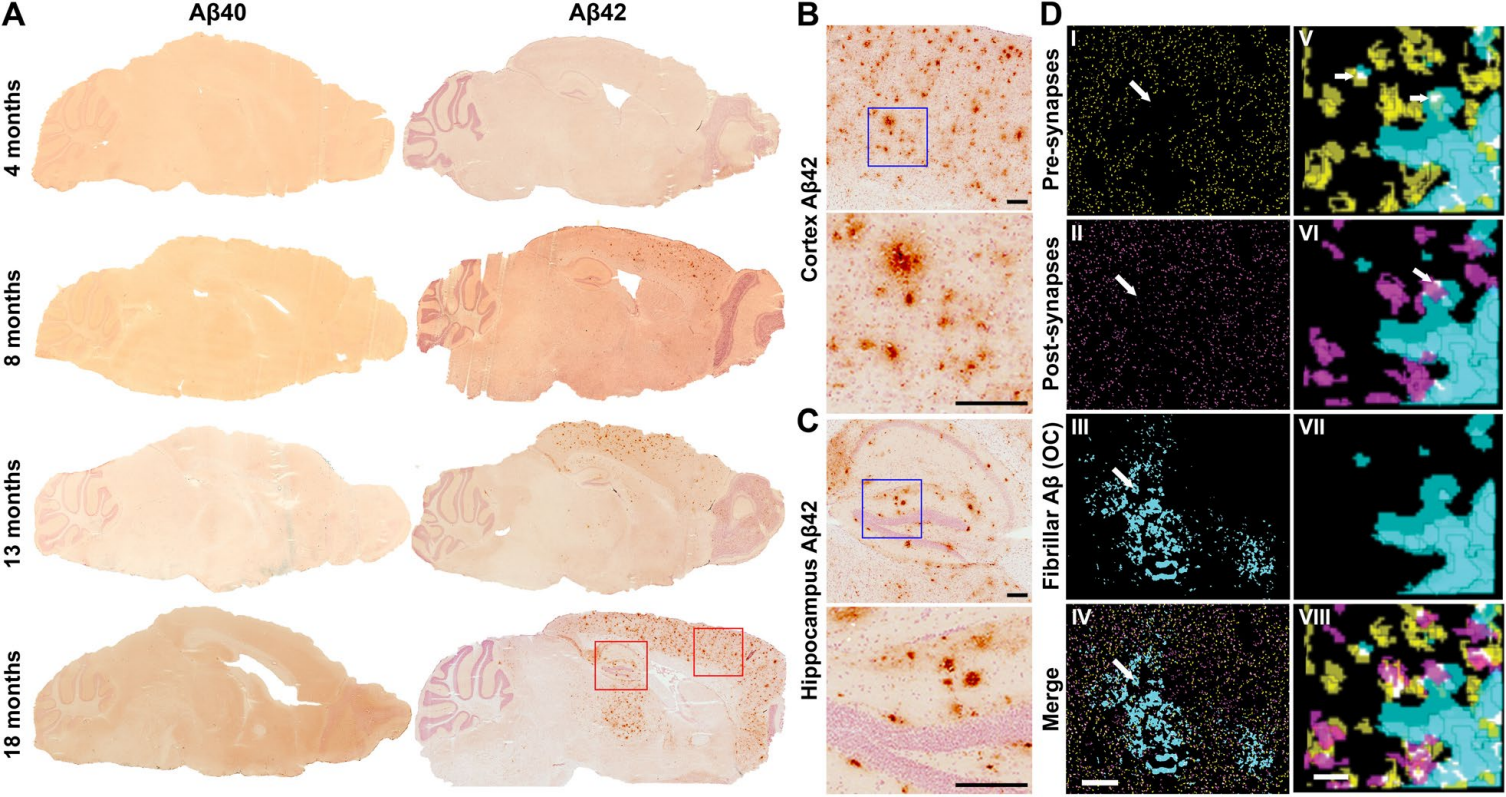
Aβ neuropathology in tg-UppSwe transgenic mice. **A** Aβ40 and Aβ42 immunostaining of tg-UppSwe mouse brain sections from different age groups. Magnifications, indicated by red squares, of cerebral cortical (**B**) and hippocampal (**C**) Aβ42 staining from 18-month-old tg-UppSwe mice. The blue squares represent the region displayed in the higher magnification images. Scale bar: 200 µm. **D** Synaptic staining of 70 nm thick serial brain sections from 18-month-old tg-UppSwe mouse stained for pre-synapses (synaptophysin; yellow), post-synapses (PSD95; magenta) and fibrillar Aβ (OC; cyan) with composite images in (IV and VIII). Synaptic staining was qualitatively reduced within plaques (arrows I–IV). Three-dimensional reconstructions (V–VIII) demonstrated Aβ staining within both pre-synapses (arrow, V) and post-synapses (arrow, VI). Panels I-IV show segmented images from a single section and V-VIII show zoomed in 3D reconstructions of five serial sections. Scale bars: 10 µm (I–IV) or 1 µm (V–VIII)

To visualize Aβ deposition over time, brain tissue sections from tg-UppSwe mice at different ages were immunostained for Aβ40 and Aβ42. In line with the low Aβ1-40 levels in FA brain extracts, Aβ40 staining was virtually absent in all age groups, except for a few diffuse deposits in hippocampus of the oldest mice (Fig. 2A). In contrast, deposition of Aβ42 was observed already at five months of age and a substantial number of plaques could be observed at eight months. Plaque pathology initially appeared in frontal cortex and then in hippocampus, increasing across the cerebral cortex until 18 months of age, when also the thalamus was affected (Fig. 2A). Differently sized Aβ42-containing plaques appeared in both cerebral cortex (Fig. 2B) and hippocampus (Fig. 2C). Array tomography analysis revealed that in the brain of 18-month-old tg-UppSwe mice, synapses appeared less dense around Aβ plaques. Moreover, aggregated Aβ co-localized with both pre- and post-synaptic markers in and around Aβ deposits (Fig. 2D).

### In vivo PET imaging of Aβ pathology

**Fig. 3:**
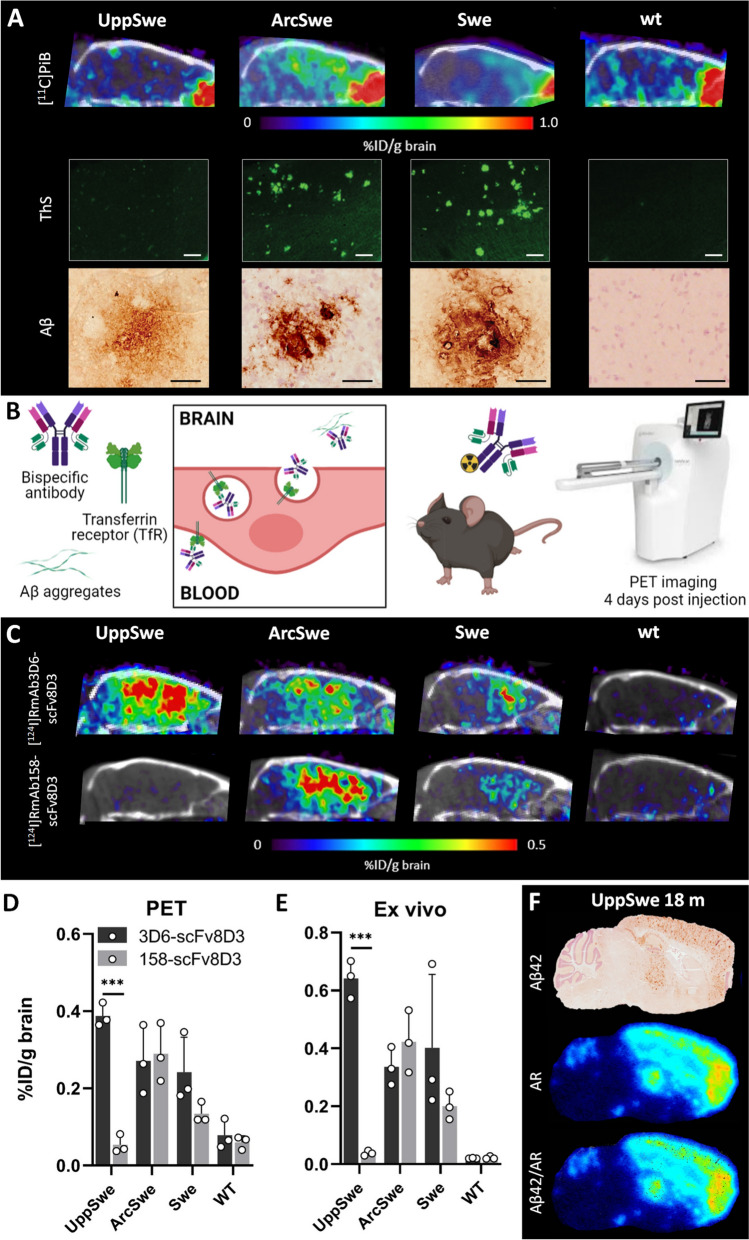
PET imaging. **A** Sagittal [^11^C]PiB-PET/CT images of 18- months-old tg-UppSwe mice in comparison with tg-ArcSwe, tg-Swe and wt mice, acquired 40–60 min after [^11^C]PiB injection, with corresponding *post mortem* brain tissue stained with thioflavin S (ThS, scale bar: 200 µm) and Aβ42 immunostaining (scale bar: 50 µm) below. **B** Bispecific Aβ antibody undergoing transcytosis across the BBB endothelium. Radiolabeled bispecific Aβ antibody ligands were used for in vivo immunoPET imaging four days after injection. **C** ImmunoPET/CT images of 18-month-old tg-UppSwe, tg-ArcSwe, tg-Swe and wt mice, injected with [^124^I]RmAb3D6-scFv8D3 or [^124^I]RmAb158-scFv8D3. **D** Quantification of immunoPET data in groups of mice (n = 3 per group). **E** Ex vivo quantification of brain radioactivity in the same mice as (**D**). **F** Aβ42 immuno-staining, ex vivo autoradiography (AR) and an Aβ42/AR merged image of brain sections from tg-UppSwe mouse injected with [^124^I]RmAb3D6-scFv8D3. ***P < 0.01

To further study the nature and structure of the Aβ pathology, 18-month-old tg-UppSwe mice were investigated by in vivo PET imaging, in comparison with tg-ArcSwe, tg-Swe and wt mice. First, mice were scanned with the small molecule amyloid PET radioligand [^11^C]PiB, considered to be the “gold standard” radioligand for in vivo PET of Aβ brain pathology. Despite the abundant Aβ pathology, as assessed with ELISA and Aβ immunostaining, no [^11^C]PiB signal was observed in the tg-UppSwe mice. As expected from previous observations, both tg-ArcSwe and tg-Swe mice displayed a clear [^11^C]PiB signal in frontal cortex (Fig. 3A), corresponding to the distribution of Aβ pathology in these transgenic mice [36]. To corroborate the [^11^C]PiB-PET results, *post mortem* brain tissue from all three models was stained with ThS, which is structurally closely related to PiB. Again, no signal was detected in tg-UppSwe mice, while both tg-ArcSwe and tg-Swe mice displayed abundant Aβ42 immunostaining of parenchymal plaques (Fig. 3A).

Next, the mice underwent immunoPET imaging with radiolabeled, brain penetrating bispecific antibodies (Fig. 3B), which have previously been demonstrated to visualize diffuse Aβ pathology in the brain of various mouse models [23, 36]. When imaged with [^124^I]RmAb3D6-scFv8D3 all three mouse lines, especially tg-UppSwe, displayed a high brain signal, with significant retention of the antibody-based radioligand in cortex, hippocampus and thalamus. In contrast, PET imaging with [^124^I]RmAb158-scFv8D3, did not generate any signal in tg-UppSwe mice. However, both tg-ArcSwe and tg-Swe mice displayed [^124^I]RmAb158-scFv8D3 retention signals comparable to those of [^124^I]RmAb3D6-scFv8D3 (Fig. 3C). The quantification of PET signals (Fig. 3D), as well as the quantification of ex vivo measured radioactivity in perfused brain tissue (Fig. 3E), verified the antibody’s retention pattern observed with PET. To closely examine the distribution of [^124^I]RmAb3D6-scFv8D3 in the tg-UppSwe mouse brain, ex vivo autoradiography was performed on fresh frozen tissue from perfused brains of PET-scanned mice. A nearly complete overlap was observed between immunostaining and antibody retention (Fig. 3F).

### Antibody reactivity and single-injection immunotherapy

**Fig. 4:**
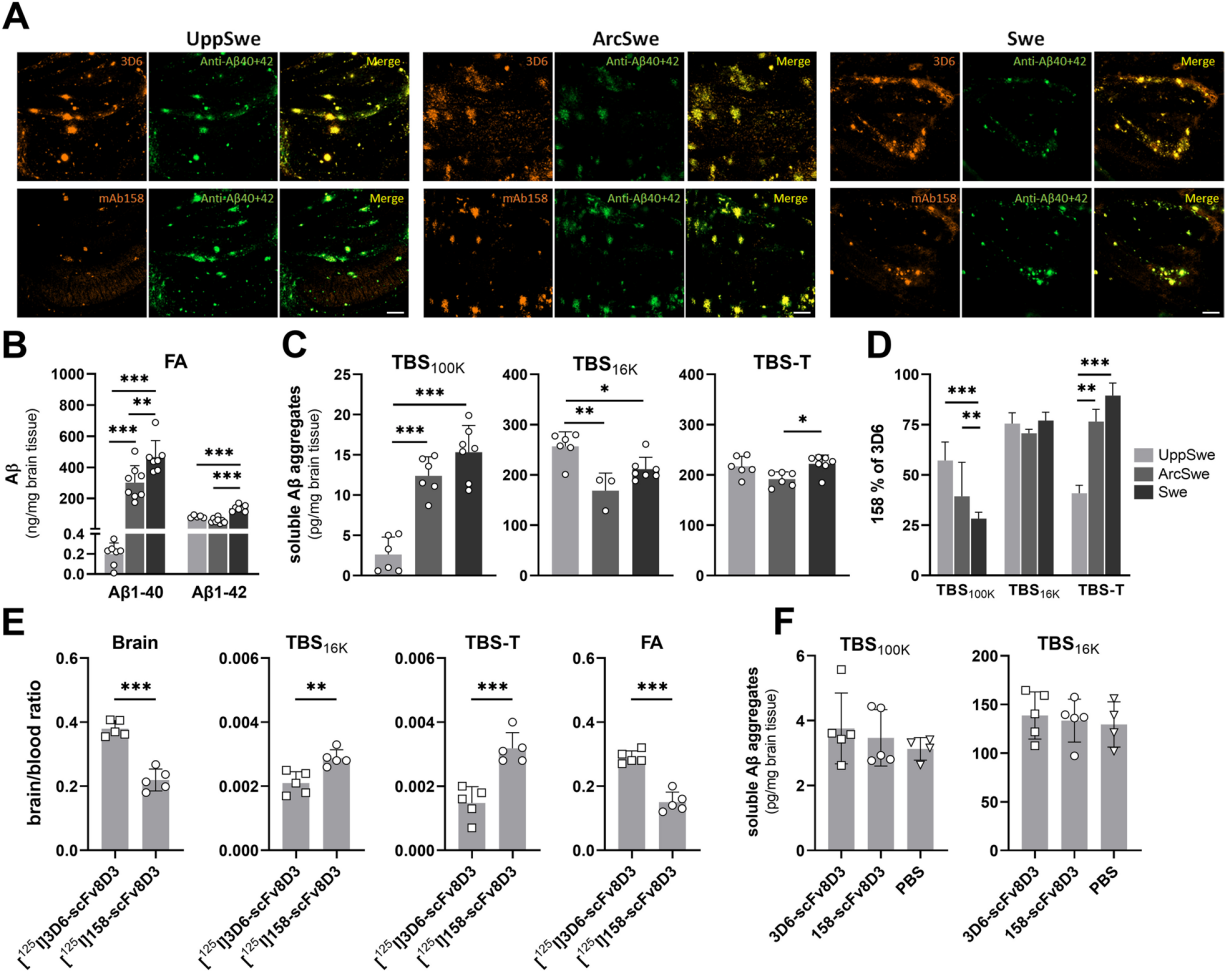
Aβ immunostaining and ELISA-based measurements of Aβ aggregates in brain tissue from tg-UppSwe, tg-ArcSwe and tg-Swe mice. **A** Immunostaining of tg-UppSwe, tgArcSwe and tg-Swe brain tissue sections (hippocampus, 40× magnification) with 3D6 (yellow, upper panel) or mAb158 (yellow, lower panel) in comparison with double staining using Aβ40 and Aβ42-specific antibodies (green). Scale bar: 200 µm. **B** ELISA quantification of total Aβ1-40 and 1-42 levels in FA brain extract.** C** 3D6-3D6 ELISA quantification of total Aβ aggregates in TBS_100K_, TBS_16K_ and TBS-T brain extracts. **D** mAb158 positive fraction of the total Aβ aggregates detected by 3D6-3D6 ELISA in TBS_100K_, TBS_16K_ and TBS-T brain extracts. **E** Distribution of [^125^I]RmAb3D6-scFv8D3 and [^125^I]RmAb158-scFv8D3, three days after administration of these bispecific antibodies at 32 nmol/kg body weight (therapeutic dose) to 18-month-old tg-UppSwe mice, expressed as a brain-to-blood radioactivity ratio in whole brain tissue (Brain) and in TBS_16K_, TBS-T and FA extracts. **F** Soluble Aβ aggregates in TBS_100K_ and TBS_16 K_ brain extracts from 18 months old tg-UppSwe mice three days after administration of a therapeutic dose (32 nmol/kg) of [^125^I]RmAb3D6-scFv8D3 or [^125^I]RmAb158-scFv8D3, in comparison with PBS. Non-significant (*ns*), *P < 0.05, **P < 0.01, ***P < 0.001

To assess 3D6 and mAb158 binding to Aβ deposits *post mortem*, brain tissue sections of the three mouse models were immunostained with either of the two antibodies and compared to Aβ40 and Aβ42 double staining. The 3D6 staining largely overlapped with the double Aβ40 + Aβ42 staining in all models. However, while tg-ArcSwe and tg-Swe mice displayed a similar staining pattern for mAb158 and Aβ40 + Aβ42, tg-UppSwe mice displayed a very faint mAb158 staining that only partly covered the surface stained with Aβ40 and Aβ42 (Fig. 4A). Sequentially extracted brain homogenates from the PET scanned mice were then analyzed by ELISA. In the FA fraction, Aβ1-40 was the dominating Aβ species in both tg-Arc-Swe and tg-Swe mice, whereas it was detected at very low levels in tg-UppSwe mice. Levels of Aβ1-42, on the other hand, were comparable between tg-UppSwe and tg-ArcSwe mice, but slightly higher in the tg-Swe model (Fig. 4B). Soluble fractions were analyzed with the 3D6-3D6 ELISA that detects aggregated Aβ from the size of a dimer and higher. In the TBS_100K_ extract, representing the most soluble fraction, tg-UppSwe mice displayed significantly lower levels of Aβ aggregates compared to tg-ArcSwe and tg-Swe mice (Fig. 4C). In the TBS_16K_ fraction, containing larger soluble aggregates and diffusely deposited aggregates, the pattern was opposite with higher levels in tg-UppSwe compared to both tg-ArcSwe and tg-Swe mice. The TBS-T fraction, with membrane associated Aβ, showed only small differences between models (Fig. 4C). When analyzing the fractions with the mAb158-3D6 ELISA, which primarily detects Aβ aggregates of larger sizes, the TBS_100K_ fraction was found to contain a higher proportion of mAb158 positive Aβ aggregates in tg-UppSwe compared to tg-ArcSwe and tg-Swe mice, while the TBS-T fraction showed the opposite pattern. In the TBS_16K_ fraction, the three models displayed no differences in the fraction of mAb158 positive soluble Aβ aggregates (Fig. 4D).

Next, we wanted to investigate whether a single antibody injection could reduce brain levels of soluble Aβ aggregates in tg-UppSwe mice, similar to what has previously been shown in the tg-ArcSwe model [38]. A therapeutic dose (32 nmol/kg body weight) of radiolabeled RmAb3D6-scFv8D3 or RmAb158-scFv8D3 was thus administered to 18-month-old tg-UppSwe mice. First, the antibody distribution in the brain was investigated by quantification of radioactivity in both whole brain and in brain extracts prepared from mice euthanized at three days after injection. Similar to the PET experiment, the [^125^I]RmAb3D6-scFv8D3 brain-to-blood concentration ratio was substantially higher than that of [^125^I]RmAb158-scFv8D3 in the whole brain (Fig. 4E). Despite its lower total brain concentration, [^125^I]RmAb158-scFv8D3 displayed a higher distribution to TBS_16K_ and TBS-T extract compared to [^125^I]RmAb3D6-scFv8D3. Approximately 95% of the antibodies were found in the FA fraction, which showed a similar distribution pattern as the whole brain, with higher concentration of [^125^I]RmAb3D6-scFv8D3 compared to [^125^I]RmAb158-scFv8D3 (Fig. 4E). However, unlike previously seen in tg-ArcSwe mice [38], none of the antibodies reduced the concentration of soluble Aβ aggregates in neither the TBS_100K_ nor the TBS_16K_ brain extracts prepared from the tg-UppSwe mice (Fig. 4F).

### ***Glial cell responses to Aβ***_***Upp***_

**Fig. 5:**
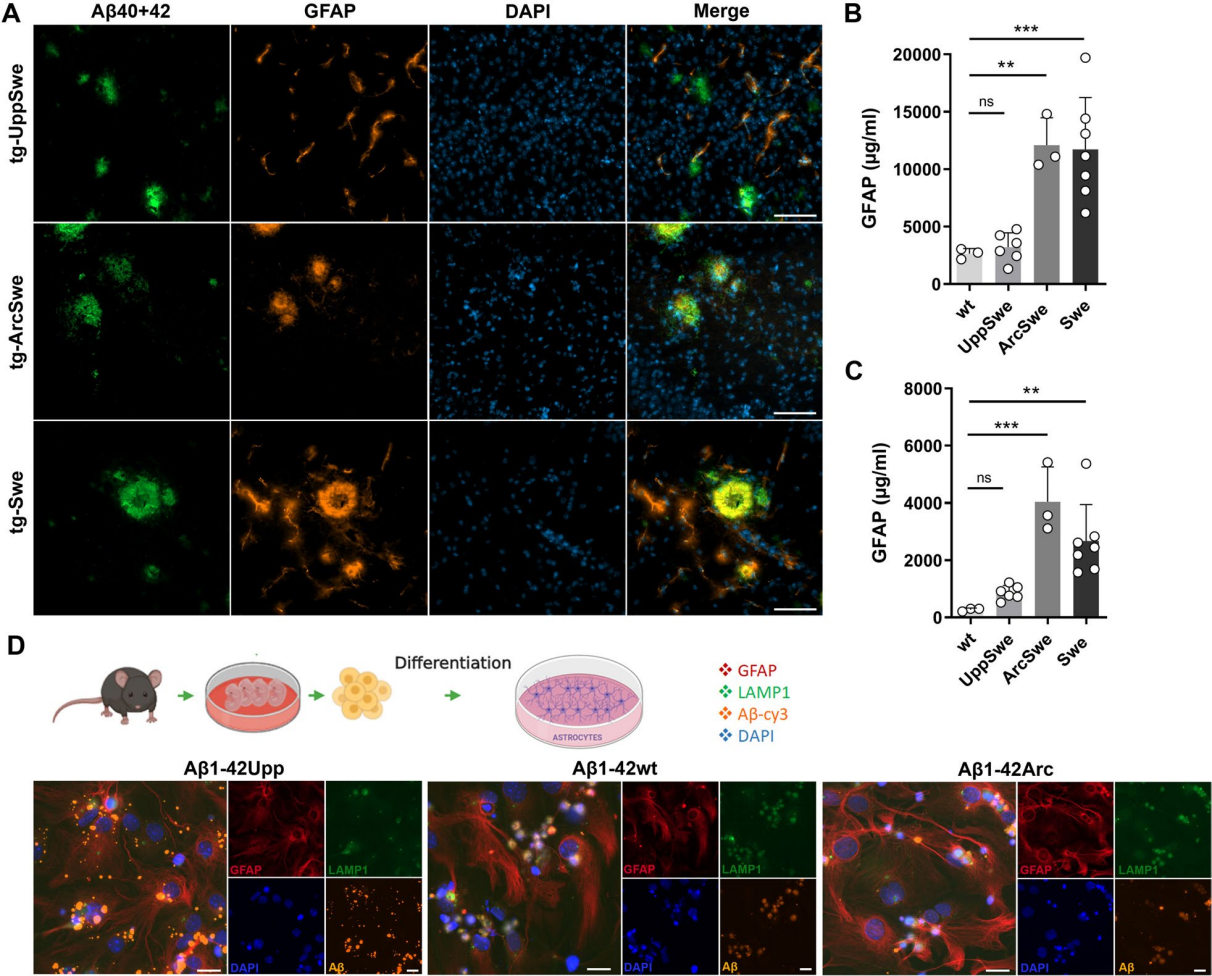
Astroglial response to Aβ. **A** Aβ40 + 42 and GFAP immunostaining of brain tissue from 18-month-old tg-UppSwe, tg-ArcSwe and tg-Swe mice. Co-localization of Aβ and GFAP was lacking in tg-UppSwe mice but was abundant in tg-ArcSwe and tg-Swe mice. Scale bar 100 µm. ELISA quantification of GFAP in TBS (**B**) and TBS-T (**C**) brain extracts from 18-months-old wt, tg-UppSwe, tg-ArcSwe and tg-Swe mice. Not significant (*ns*), **P < 0.01, ***P < 0.001. **D** Schematic description of the procedure to establish primary astrocyte monocultures originating from the cerebral cortices of embryonic mouse brain. Primary astrocyte cultures were exposed to sonicated, Cy3-labeled fibrils of synthetic Aβ_Upp_, Aβ_Arc_ or Aβ_wt_ (all Aβ1-42). Cells were stained for GFAP, LAMP-1 and cell nuclei (Dapi). While Aβ_Upp_ clustered on the surface of cells, Aβ_Arc_ and Aβ_wt_ appeared to be phagocytosed to a larger extent. Scale bar: 20 µm

In brain tissues from 18-month-old tg-UppSwe mice, astrocytes stained for glial fibrillary acidic protein (GFAP) did not co-localize with Aβ to a large extent, while both tg-ArcSwe and tg-Swe mice of the same age displayed intense GFAP staining in close proximity to the Aβ-staining (Fig. 5A). When quantified by ELISA, tg-UppSwe mice showed lower GFAP levels in TBS soluble brain extracts (Fig. 5B) and only a modest increase in TBS-T extracts compared to wt mice (Fig. 5C). However, both tg-ArcSwe and tg-Swe mice displayed elevated levels of GFAP in both TBS and TBS-T brain extracts (Fig. 5B, C).

To further investigate the astrocytic response to Aβ aggregates, primary mouse astrocyte cultures were exposed to sonicated, fluorophore-labeled fibrils of synthetic Aβ_Upp_ (present in tg-UppSwe), Aβ_Arc_ (present in tg-ArcSwe) or Aβ_wt_ (present in tg-Swe) (Fig. 5D). The Aβ_Upp_ fibrils were readily visible in the culture but seemed to cluster on the cell surface rather than being phagocytosed. This was supported by the lack of co-localization between Aβ_Upp_ and the lysosomal-associated membrane protein 1 (LAMP-1). In contrast, both Aβ_Arc_ and Aβ_wt_ were found in the vicinity of LAMP-1 positive structures, suggesting that they had been taken up by the cells (Fig. 5D).

**Fig. 6:**
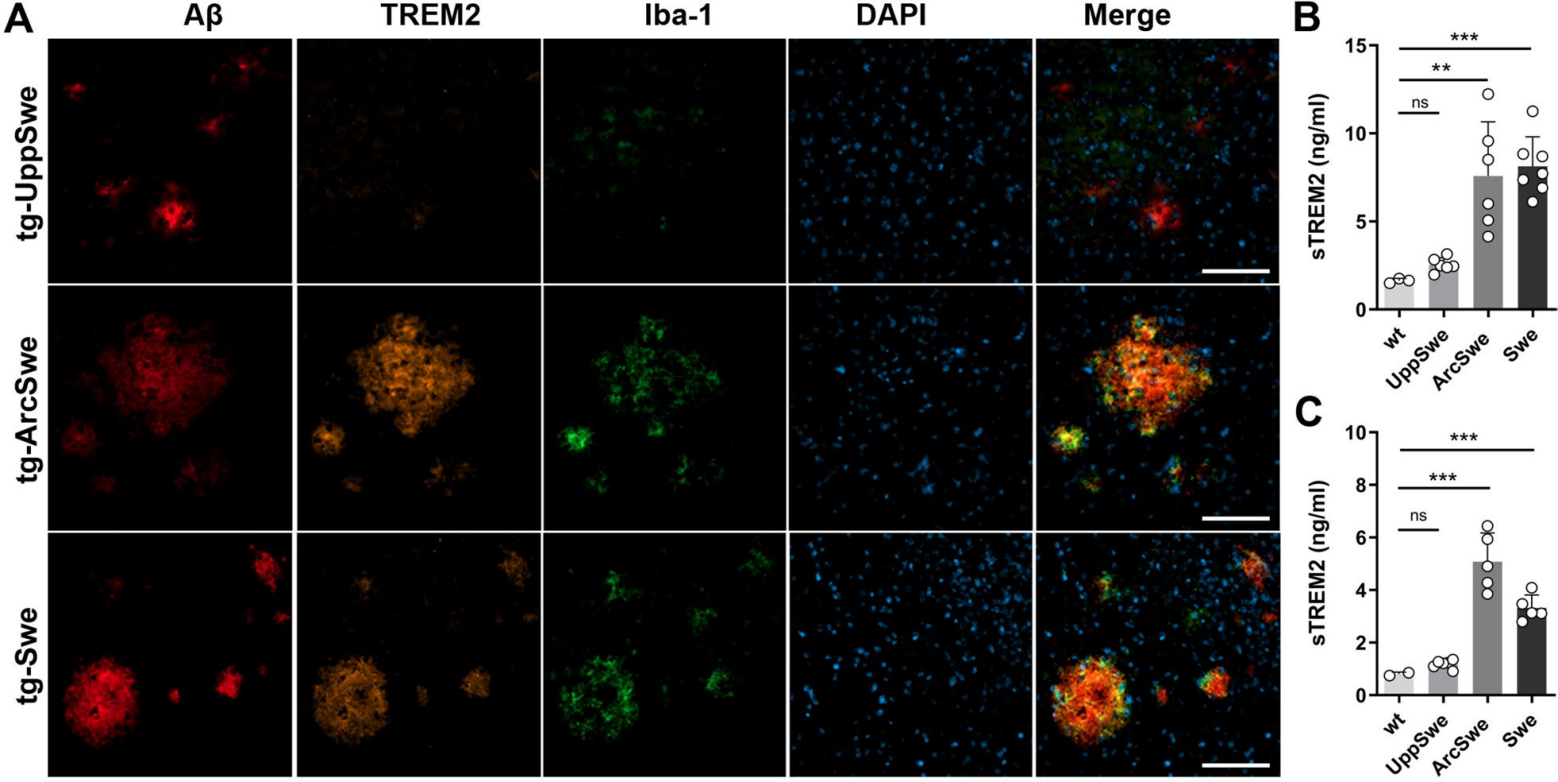
Microglial response to Aβ. **A** Aβ, TREM2 and Iba-1 immunostaining of brain tissue from 18-months-old tg-UppSwe, tg-ArcSwe and tg-Swe mice. Scale bar: 100 µm. ELISA quantification of soluble TREM2 in TBS_16K_ (**B**) and TBS-T (**C**) brain extracts from 18-months-old wt, tg-UppSwe, tg-ArcSwe and tg-Swe mice. Not significant (*ns*), *P < 0.05, **P < 0.01, ***P < 0.001

Immunostaining of Aβ deposits in combination with the microglial markers TREM2 and Iba-1 revealed a similar pattern as for astrocytes. In 18-month-old tg-UppSwe mice, microglial staining did not co-localize with Aβ deposits, while both tg-ArcSwe and tg-Swe mice of the same age showed robust staining of both microglial markers in the vicinity of Aβ deposits (Fig. 6A). The microglial response to Aβ pathology was further evaluated by ELISA analysis of soluble TREM2 (sTREM2) in TBS_16K_ and TBS-T brain extracts from the three models and age matched wt mice. Only background sTREM2 levels were detected in tg-UppSwe mice, while both tg-ArcSwe and tg-Swe mice had elevated sTREM2 in both TBS_16K_ and TBS-T (Fig. 6B, C).

## Discussion

Historically, APP transgenic mice have proven useful to further our understanding of the pathological processes of AD, as well as for the development of novel therapeutic and diagnostic approaches. Notably, the Aβ protofibril selective monoclonal antibody mAb158 [2], the murine parent antibody of lecanemab, was developed and evaluated in our laboratory using the in-house developed tg-ArcSwe mouse model to assess therapeutic efficacy [19]. The tg-ArcSwe model has been particularly valuable for studies of antibody target engagement, as it presents Aβ pathology closely resembling that in sporadic AD, both in terms of structure [42] and biochemical properties [29]. Here we demonstrate that the newly generated tg-UppSwe mouse model, designed in the same way as tg-ArcSwe, displays all the primary pathological features of the *APPUpp* deletion that causes early-onset familial AD [27]. Similar to human *APPUpp* carriers, tg-UppSwe mice show an increased Aβ promoting β-secretase cleavage in combination with a suppressed anti-amyloidogenic α-secretase cleavage. Notably, tg-UppSwe mice carry the *Swedish APP* mutation, which enhances β-secretase cleavage. Still, β-secretase cleavage was markedly higher compared to both tg-ArcSwe and tg-Swe mice, which both carry the *Swedish APP* mutation. The potential impact of *APPUpp* on γ-secretase cleavage has not been studied previously. Here, we found that the soluble brain extract from young tg-UppSwe mice have an Aβ42/Aβ40 ratio of around 1:10. This suggests that *APPUpp* does not significantly affect γ-secretase cleavage, in contrast to the *APP* mutations near the C-terminal end of the Aβ domain and the presenilin (*PSEN*) mutations, which cause a shift from Aβ40 to Aβ42 (reviewed in [7]). Similar to the *APPUpp* patient brain [27], the tg-UppSwe mouse brain features plaque pathology dominated by Aβ42, with only minimal Aβ40 contribution. Tg-UppSwe mice lack expression of Aβ_wt_ and, although they harbor only one copy of *hAPP*, they display an early and extensive deposition of small diffuse plaques that appear in frontal cortex already at 6–8 months and later across the cerebral cortical and hippocampal regions, with deposition also in thalamus at 18 months of age. Thus, this aggressive Aβ42 pathology can likely be attributed to a rapid Aβ aggregation [27] rather than an overproduction of Aβ_Upp_1-42.

PET imaging was employed to further investigate the structure of Aβ pathology in the living mouse brain, using three different radioligands. The clinically used amyloid radioligand [^11^C]PiB, which binds to dense core plaques, did not detect Aβ pathology in tg-UppSwe mice. This is partly consistent with the modest [^11^C]PiB signal seen in patients carrying the *APPUpp* deletion despite substantial Aβ deposition in the brain [27]. As seen in prior studies [3, 36], both tg-ArcSwe and tg-Swe mice showed signals corresponding to areas rich in ThS positive Aβ pathology. The strongest [^11^C]PiB-PET signal was observed in the tg-ArcSwe model, known to develop Aβ fibrils structurally similar to those in sporadic AD brains [29, 42]. In contrast to [^11^C]PiB, immunoPET with [^125^I]RmAb3D6-scFv8D3 produced a strong signal in tg-UppSwe mice, which appeared even stronger than that in tg-ArcSwe and tg-Swe mice. Although this difference was not significant, the result is striking as the total level of Aβ (i.e. Aβ1-40 + Aβ1-42), as determined by ELISA, was much lower in tg-UppSwe compared to tg-ArcSwe and tg-Swe mice. ImmunoPET imaging conducted four days post injection of the antibody ligand requires that the antigen is immobile in the tissue, suggesting that mostly diffuse and fibrillar deposited Aβ will be detected. This was confirmed by the observation that approximately 95% of [^125^I]RmAb3D6-scFv8D3 was found in the FA fraction of tg-UppSwe brain *post mortem*, supposedly bound to the diffuse plaque pathology, whereas only a small fraction of the antibody was detected in the TBS and TBS-T soluble brain fractions. We speculate that the less compact structure of the tg-UppSwe plaques provides a better accessibility for the antibody and a higher number of available binding sites compared to the dense plaques found in tg-ArcSwe and tg-Swe mice.

Surprisingly, PET imaging with the Aβ protofibril selective antibody [^125^I]RmAb158-scFv8D3 did not detect Aβ in tg-UppSwe mice, while both tg-ArcSwe and tg-Swe mice displayed high signals, suggesting a structural difference in Aβ aggregates between the mouse models. It has been reported that the conversion from soluble Aβ protofibrils to fibrils requires a conformational change of the aggregate into a cross-β structure [33]. As mAb158 preferentially binds to soluble Aβ aggregates [30, 35], we hypothesize that fibrillization of Aβ_Upp_ leads to a structural change, where the mAb158 epitope becomes inaccessible in fibrillar tg-UppSwe Aβ deposits. The rapid aggregation of Aβ_Upp_ could explain why tg-UppSwe mice had the highest proportion of mAb158 positive Aβ aggregates in the most soluble fraction (TBS_100K_), indicative of a high proportion of large soluble aggregates before formation of insoluble fibrils. Interestingly, the absolute levels of TBS_100K_ Aβ aggregates were extremely low in tg-UppSwe compared to tg-ArcSwe and tg-Swe mice, suggesting that soluble Aβ aggregates in tg-UppSwe mice are rapidly converted from mAb158 positive, soluble Aβ protofibrils into fibrillar Aβ, while adopting a structure that is poorly detected by the Aβ protofibril selective mAb158. This is in line with the structural difference of Aβ_Upp_ compared to Aβ_wt_ fibrils proposed by previous preliminary cryo-EM analyses [27] and would explain the low immunoPET signal seen with [^124^I]RmAb158-scFv8D3 in tg-UppSwe mice. The low levels of soluble Aβ aggregates could also explain why neither RmAb158-scFv8D3 nor RmAb3D6-scFv8D3 demonstrated a therapeutic effect on soluble Aβ aggregates in the TBS_100K_ fraction. These results are reminiscent to those when treating *App*^*NL-G-F*^ mice with a single injection of RmAb158-scFv8D3 [6, 31].

In AD, Aβ pathology is accompanied by tau pathology and extensive neuroinflammation. No tau pathology was observed in tg-UppSwe mice. In addition, and contrary to tg-ArcSwe and tg-Swe mice, very little activation of microglia or astrocytes was detected around plaques in the tg-UppSwe brain. Moreover, cell-based experiments illustrated that astrocytes in culture did not internalize Aβ_Upp_, whereas both Aβ_wt_ and Aβ_Arc_ were readily taken up by the cells. The observations discussed above suggest at least two potential explanations for the lack of glial involvement—low levels of diffusible Aβ oligomers or a structural difference of the full-sized fibrils. We have previously reported that cultured astrocytes effectively ingest Aβ_wt_ protofibrils as well as sonicated fibrils. We have, however, noticed that larger fibrils are not easily phagocytosed by the astrocytes. Moreover, astrocytes process Aβ_wt_ differentially depending on its aggregation state. While the cells easily degrade monomeric proteins, larger oligomers and sonicated fibrils are accumulated intracellularly [15, 16, 37, 43]. These studies suggest that Aβ protofibrils or sonicated fibrils of Aβ_wt_ can be processed by and activate astrocytes, while monomers and full-sized Aβ fibrils do not have the same effect. Here, Aβ_Upp_ fibrils were not taken up by astrocytes to the same extent as Aβ_wt_ or Aβ_Arc_ fibrils. It remains to be investigated whether this reduced uptake is due to a difference in the structure of the sonicated fibrils or to re-fibrillization of Aβ_Upp_ fibrils into larger structures that cannot be internalized by the astrocytes due to their size. In addition, we have previously observed synergistic effects of astrocytes and microglia in the processing of Aβ aggregates [32]. Hence, a reduced astrocytic uptake of Aβ_Upp_ fibrils could lead to decreased cytokine release, which could contribute to a reduced activation of microglia around the plaques.

Previous studies have suggested a direct link between oligomeric Aβ and microglial activation via the TREM2 receptor [18], and that this interaction can be influenced by different cleavages and post-translational modifications that could affect Aβ aggregation [10]. In the current study, we observed a lack of microglial staining associated with Aβ deposits in tg-UppSwe mice, while both tg-ArcSwe and tg-Swe mice displayed abundant staining of both the general microglial marker Iba-1 and the more AD specific marker TREM2. The ELISA analysis of sTREM2 confirmed this pattern, with sTREM2 levels in tg-UppSwe in the same range as observed in wt mice, while both tg-ArcSwe and tg-Swe mice had elevated levels. Importantly, TREM2 in combination with TAM receptors, has been implicated in the process of compacting Aβ deposits into dense plaques [8]. The absence of TREM2 upregulation in tg-UppSwe mice thus suggests that Aβ_Upp_ oligomers do not interact with TREM2 to induce compacting of Aβ plaques, resulting in a widespread, diffuse Aβ pathology with small deposits in all major brain areas. While this could be due to a difference in structure of Aβ_Upp_ oligomers, we hypothesize that the extremely rapid conversion from oligomers to fibrils may also be a plausible explanation for the lack of glial involvement in the tg-UppSwe brain. The degree of microglial activation has not been studied in human *APPUpp* mutation carriers and a direct comparison with the human situation would be further complicated by the fact that the only *APPUpp* mutation carrier who has come to autopsy also has a *TREM2* mutation [28], which in itself may have an effect on microlgial activation and distribution around Aβ deposits.

In conclusion, the novel tg-UppSwe mouse model that harbors the *APPUpp* mutation recapitulates the pathological mechanisms of human mutation carriers. We here demonstrate that the aggressive aggregation of Aβ_Upp_ results in low levels of soluble Aβ oligomers, but abundant and widespread diffuse Aβ deposits that differ structurally from aggregates of Aβ_wt_ and Aβ_Arc_, as visualized by in vivo PET imaging. Additionally, we show that different Aβ structures are distinctly recognized by individual Aβ antibodies. The unique properties of Aβ_Upp_ result in minimal glial activation and, as a consequence, fewer compacted dense amyloid plaques. The tg-UppSwe mouse thus serves as a valuable model for studying various aspects of Aβ plaque formation as well as glial involvement in AD pathogenesis. These findings could have important implications for the continued development of disease-modifying therapies for AD.

### Supplementary Information

**Additional file 1:** **Figure S1.** Schematic description of homogenization and sequential extraction of the mouse brain tissue, resulting in the TBS16K, TBS100K, TBS-T and FA fractions. **Figure S2. APP expression in UppSwe transgenic mice.** **A.** Number of hAPP copies in the different tg-mouse lines. **B.** MSD immunoassay analysis showing lower concentration of sAPPα in TBS brain extracts of tg-UppSwe mice compared to tg-ArcSwe. **C.** Western blot analysis demonstrating that with the 2B3 antibody, sAPPα was barely detectable in the tg-UppSwe brains, while sAPPβ signals detected with the Sw192 antibody were strong. Total sAPP was detected with the 22C11 antibody and β-actin was used as loading control. **D.** Quantifications of the western blot analyses with total normalization to total sAPP. **E.** Full western blot membranes from (**C**). **Figure S3. Body weights of transgenic tg-Swe, tg-ArcSwe and tg-UppSwe in comparison with wt mice at different ages.** Female mice of all transgenic lines displayed a significantly reduced body weight compared to wt mice at both young (8-10 months) and old (17-19 months) age. For male mice, only old tg-Swe mice displayed a reduced weight. Tg-Swe mice aged 8-10 months were not accessible. **P < 0.01, ***P < 0.001.

## Data Availability

The datasets used and/or analysed during the current study are available from the corresponding author on reasonable request.
