# Supplementary material for: Altered amyloid-β structure markedly reduces gliosis in the brain of mice harboring the Uppsala APP deletion
Source: Acta Neuropathol Commun. 2024 Feb 5;12:22. doi: 10.1186/s40478-024-01734-x (PMC10845526; doi:10.1186/s40478-024-01734-x)
Supplement: Supplementary file 1 — Additional file 1: Figure S1. Schematic description of homogenization and sequential extraction of the mouse brain tissue, resulting in the TBS16K, TBS100K, TBS-T and FA fractions. Figure S2. APP expression in UppSwe transgenic mice. A. Number of hAPP copies in the different tg-mouse lines. B. MSD immunoassay analysis showing lower concentration of sAPPα in TBS brain extracts of tg-UppSwe mice compared to tg-ArcSwe. C. Western blot analysis demonstrating that with the 2B3 antibody, sAPPα was barely detectable in the tg-UppSwe brains, while sAPPβ signals detected with the Sw192 antibody were strong. Total sAPP was detected with the 22C11 antibody and β-actin was used as loading control. D. Quantifications of the western blot analyses with total normalization to total sAPP. E. Full western blot membranes from (C). Figure S3. Body weights of transgenic tg-Swe, tg-ArcSwe and tg-UppSwe in comparison with wt mice at different ages. Female mice of all transgenic lines displayed a significantly reduced body weight compared to wt mice at both young (8-10 months) and old (17-19 months) age. For male mice, only old tg-Swe mice displayed a reduced weight. Tg-Swe mice aged 8-10 months were not accessible. **P < 0.01, ***P < 0.001. [file 40478_2024_1734_MOESM1_ESM.docx]

**
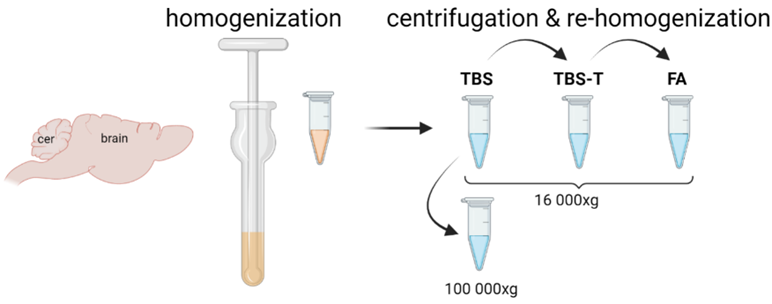
Supplementary Information**

**Figure 1**. Schematic description of homogenization and sequential extraction of the mouse brain tissue, resulting in the TBS_16K_, TBS_100K_, TBS-T and FA fractions

**
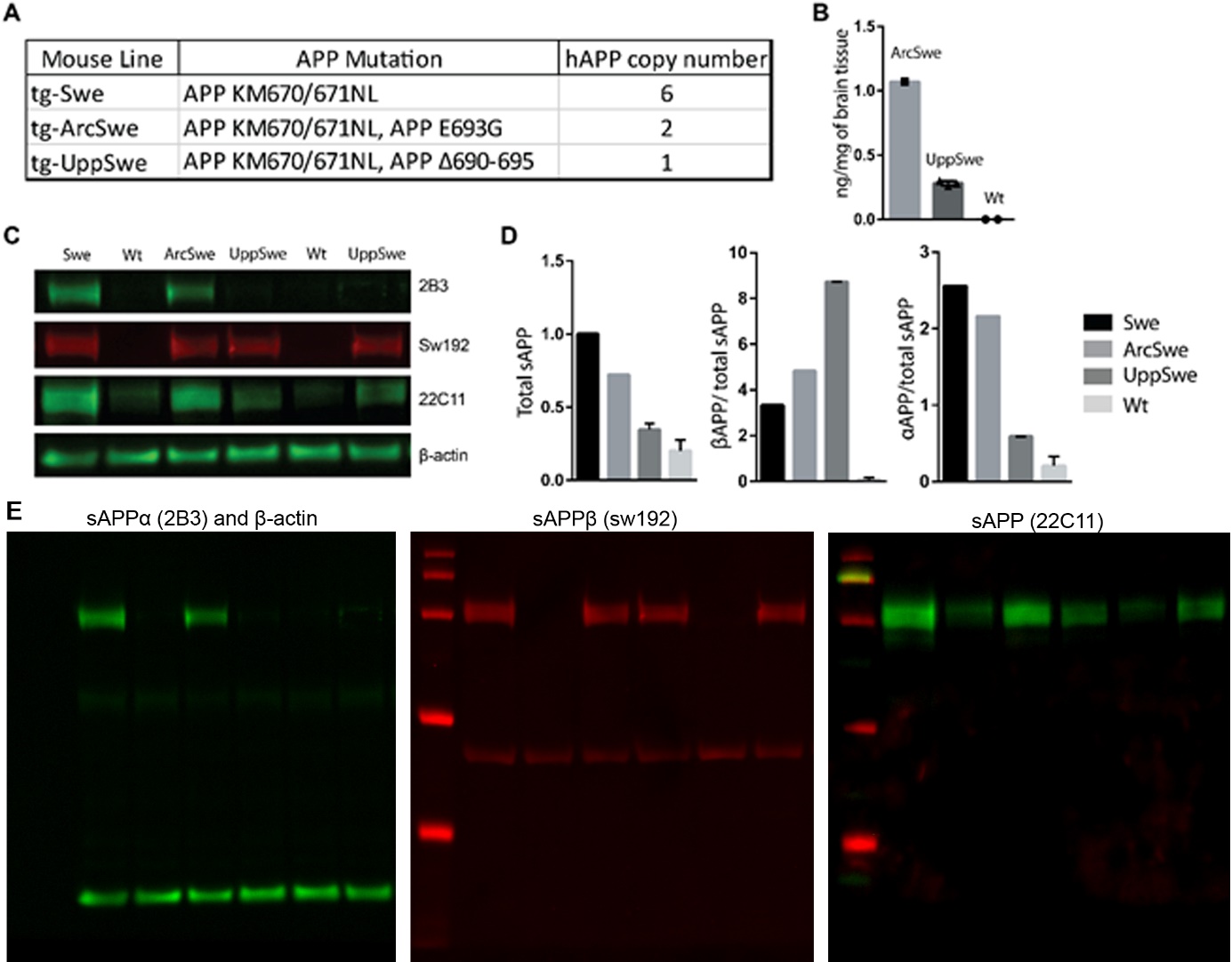
**

**Figure 2. APP expression in UppSwe transgenic mice. A.** Number of hAPP copies in the different tg-mouse lines. **B.** MSD immunoassay analysis showing lower concentration of sAPPα in TBS brain extracts of tg-UppSwe mice compared to tg-ArcSwe**.** **C.** Western blot analysis demonstrating that with the 2B3 antibody, sAPPα was barely detectable in the tg-UppSwe brains, while sAPPβ signals detected with the Sw192 antibody were strong. Total sAPP was detected with the 22C11 antibody and β-actin was used as loading control. **D.** Quantifications of the western blot analyses with total normalization to total sAPP. **E.** Full western blot membranes from (**C**).


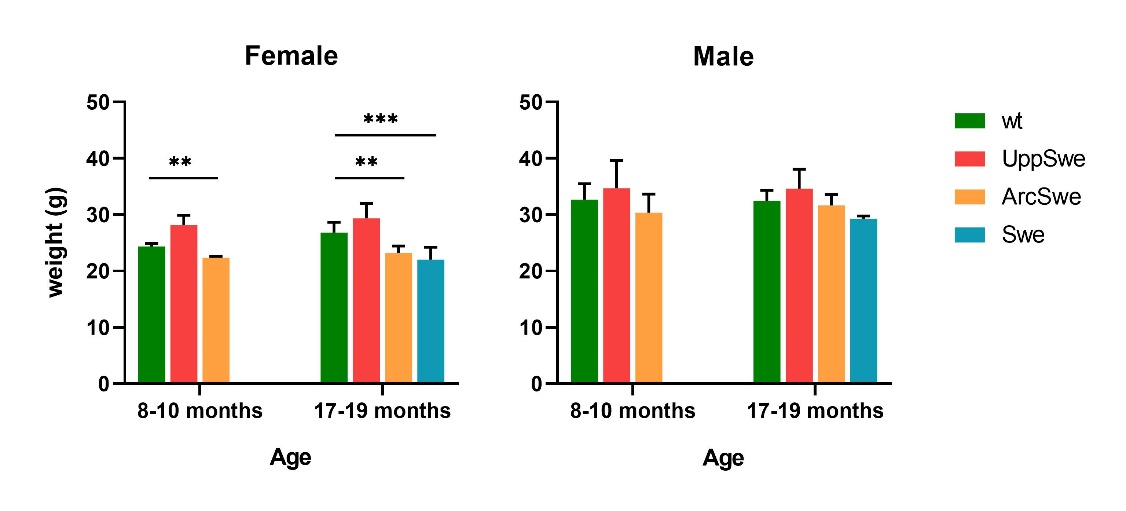


**Figure 3. Body weights of transgenic tg-Swe, tg-ArcSwe and tg-UppSwe in comparison with wt mice at different ages.** Female mice of all transgenic lines displayed a significantly reduced body weight compared to wt mice at both young (8-10 months) and old (17-19 months) age. For male mice, only old tg-Swe mice displayed a reduced weight. Tg-Swe mice aged 8-10 months were not accessible. ***P*<0.01, ****P*<0.001.
